# Recruitment of neutrophils by granulocyte colony-stimulating factor in cancer patients undergoing immunotherapy: the good, the bad, and the unknown

**DOI:** 10.3389/fimmu.2026.1718838

**Published:** 2026-02-04

**Authors:** Irina Krykbaeva, Karishma Vijay Rupani, Johnson M. Liu

**Affiliations:** Division of Hematology and Medical Oncology, Icahn School of Medicine at Mount Sinai, New York, NY, United States

**Keywords:** chemo-immunotherapy, G-CSF, immune checkpoint inhibitor, neutrophil recruitment, tumor microenvironment, tumor-associated neutrophil

## Abstract

Granulocyte-colony stimulating factor (G-CSF) is a cornerstone of supportive care in oncology, widely used to prevent chemotherapy-induced neutropenia and maintain dose intensity and treatment schedule. While G-CSF has been shown to be safe and effective in the context of chemotherapy, its effects in patients receiving chemo-immunotherapy with immune checkpoint inhibitors (ICIs) are less clear. There is a growing body of preclinical data to suggest that G-CSF negatively impacts the tumor microenvironment in mice and may contribute to tumor growth and metastasis. Consequently, as chemo-immunotherapy regimens have become standard across solid malignancies, the immunologic consequences of G-CSF in this setting have come under renewed scrutiny. G-CSF is a pleiotropic cytokine, and one of its most prominent functions is to stimulate the proliferation and survival of neutrophils, which in the tumor microenvironment may exert both anti- and pro-tumorigenic roles. G-CSF stimulated neutrophils have been shown to contribute to the anti-tumor immune response via upregulation of unconventional T cells, maintenance of interferon gamma- based immunity, and direct tumor cell killing in some models. Other models, however, have shown that cytokine mixes containing G-CSF can polarize neutrophils towards a pro-tumorigenic state, promoting suppression of CD8+ T cells, creating an immunosuppressive cytokine milieu, and increasing metastatic potential. Ultimately, its effects appear to be dependent on the underlying immune state and composition of the tumor microenvironment. While preclinical data suggest that G-CSF may be blunting the efficacy of checkpoint blockade via its modulation of the neutrophil compartment in specific models, clinical data in the immunotherapy era remain limited and largely retrospective. Studies done primarily in lung cancer patients showed neither a clear survival benefit nor an increased mortality risk. In this review, we synthesize the preclinical and clinical literature examining G-CSF in solid tumor oncology, with a focus on its interaction with neutrophils and immune checkpoint inhibition. We highlight key mechanistic insights, emerging clinical signals, gaps in evidence, and ultimately emphasize the importance of adhering to strict, consensus guideline-based use of G-CSF during chemo-immunotherapy.

## Introduction

Granulocyte colony stimulating factor (G-CSF) has long had a role in cancer treatment. G-CSF stimulates granulopoiesis and mobilization of granulocytes, most prominently neutrophils. It is commonly used to treat chemotherapy-induced neutropenia in patients with solid malignancies. As newer therapies are introduced, treatment with combinations of chemotherapy and immunotherapy has become more widespread. The role of G-CSF in this context is less clear. Although there is no definitive clinical evidence that G-CSF affects prognosis in the context of immune checkpoint inhibition, an abundance of preclinical data as well as a few clinical studies suggest that G-CSF may worsen outcomes in this setting. Here we highlight both the preclinical and clinical literature exploring the role of G-CSF in solid malignancies.

## The role of G-CSF in neutrophil recruitment within the tumor microenvironment: preclinical data

### Neutrophil heterogeneity within the TME reflects both pro-tumorigenic and anti-tumorigenic functional states

Neutrophils are the most abundant immune cell type in the peripheral blood and are responsible for antimicrobial immunity. In the context of malignancy, an elevated neutrophil-to-lymphocyte ratio is associated with a poor prognosis (1–3). However, neutrophils have been shown to have both tumor-promoting and tumor-suppressive functions. As high throughput sequencing techniques have become widely utilized to interrogate the tumor microenvironment (TME), they have revealed considerable heterogeneity within the neutrophil population. Neutrophils present within the tumor are often phenotypically different from those found within the peripheral blood and are called tumor-associated neutrophils (TANs). Single cell RNA sequencing has identified numerous distinct TAN subsets, each with their own gene expression signatures. These subtypes are associated with a spectrum of activation states that dictates their role in the tumor microenvironment (4–7). Although neutrophil activation exists on a spectrum, traditionally, the ‘N1’ state denotes acquisition of anti-tumor functions, whereas the ‘N2’ state refers to acquisition of pro-tumorigenic functions (4, 8, 9). N2 TANs are phenotypically and functionally similar to a subset of myeloid-derived suppressor cells (MDSC) that have been classified as polymorphonuclear myeloid-derived suppressor cells (PMN-MDSCs). PMN-MDSCs are a heterogenous population of both mature and immature circulating granulocytic myeloid cells that are found in the peripheral blood and are characterized by their immunosuppressive functions, in particular their ability to impair CD8^+^ T cell responses (10). While TANs are classified primarily in relation to their effect on tumor growth and PMN-MDSCs by their effect on the anti-tumor immune response, in practical terms it is very difficult to distinguish between the two populations. As of now there are no markers that can reliably differentiate between them, and there is debate in the literature as to whether N2 TANs and PMN-MDSCs constitute overlapping cell populations. Regardless, both N2 TANs and PMN-MDSCs have been shown to have tumor-promoting characteristics. This review will broadly focus on tumor infiltrating neutrophils, acknowledging that it may be challenging to further classify relevant populations into specific TAN or PMN-MDSC categories (10, 11).

Anti-tumor neutrophils have been shown to participate in tumor cell killing via antibody-dependent cellular cytotoxicity (ADCC), degranulation, and T cell activation among other mechanisms (12–14). One study found that human neutrophils release neutrophil elastase on degranulation that specifically kills tumor cells in multiple human cancer cell lines and xenograft mouse models (12). Neutrophils can also participate in ADCC via their FcαR receptors. G-CSF-mobilized immature neutrophils have been shown to effectively kill tumor cells via engagement of the FcαR receptor with tumor specific antigens in breast cancer and lymphoma cell lines as well as in transgenic mice (13). Furthermore, they have been shown to facilitate tumor cell death via autophagy in human mammary carcinoma cell lines when incubated with tumor antigen specific FcαR1 bispecific antibodies (15). Neutrophil engaging bispecific antibodies are being developed (16). There is also data to suggest that peripheral blood neutrophils can induce cell cycle arrest and apoptosis in a non-small cell lung cancer (NSCLC) cell line via the FasL pathway (17, 18). Neutrophils have also been shown to promote CD8+ T cell responses via suppression of IL-17 signaling in a murine lung cancer model (19). Colocalization of neutrophils with CD8+ T cells in colorectal cancer tissue samples was correlated with a favorable prognosis and was hypothesized to promote CD8+ T cell activation and a central memory T cell phenotype (20).

Conversely, pro-tumorigenic neutrophils promote upregulation of immune checkpoints, secrete immunosuppressive cytokines, and recruit regulatory T cells to protect the tumor against T cell based immunity (21–23). Tumor-derived neutrophils, or TANs, were shown to upregulate the cMET/STAT3/PDL1 pathway via IL-10 in human lung adenocarcinoma cells and create a positive feedback loop promoting tumorigenesis and metastasis in a corresponding murine model (21). Although there is controversy regarding whether human neutrophils produce IL-10, the authors showed elevated IL-10 levels via ELISA in the supernatants of TANs collected from lung cancer patients. They also found a positive correlation between TAN expression and poor prognosis in these patients (21). Another study found that immunosuppressive Arg1^+^PDL1^+^ neutrophils suppress CD8^+^ T cell proliferation and promote growth of brain metastasis in a murine model of breast cancer (22). TANs were also found to recruit macrophages and regulatory T cells to tumors, resulting in larger tumors and more metastasis, in a study with both human cell lines and murine models of hepatocellular carcinoma (HCC) (23). N2-like neutrophils have also been shown to contribute to remodeling of the extracellular matrix (ECM) to facilitate tumor invasion and are crucial for the establishment of micro-metastases (24). Neutrophils can secrete neutrophil extracellular traps (NETs), which are web-like structures composed of DNA and granule proteins. Although NETs have been shown to have some anti-tumorigenic functions, the majority of the literature suggests that NETs are involved in tumor progression. They have been shown to have crucial roles in early metastasis using patient samples, human cell lines, and mouse models, and can activate dormant cancer cells by degrading the ECM in mice (25–27). They can also act as a physical barrier against T cells and contribute to tumor resistance to immune checkpoint blockade (28, 29).

In early tumor development, neutrophils are recruited from the periphery and are thought to exhibit more of an N1-like profile. As the tumor grows, neutrophils are influenced by the changing TME and intra-tumoral conditions such as hypoxia and chronic inflammation, which induce reprogramming towards an N2-like state (4, 30, 31). Reprogramming occurs via complex signaling networks within the TME directed by a milieu of tumor-derived cytokines. G-CSF is known to be a key regulator of neutrophil survival and has been shown to be an active player in tumor-associated neutrophil regulation.

### G-CSF is a pleiotropic cytokine that exerts immunomodulatory effects on the TME and is associated with poor outcomes

G-CSF is a growth factor that promotes survival, proliferation, and maturation of neutrophils in the bone marrow. It is a glycoprotein that can be secreted by the majority of cell types within the body. Under steady state conditions, G-CSF is produced by bone marrow stromal cells to stimulate granulopoiesis to mature neutrophils. Under stress conditions such as infection, inflammation, or injury, the release of inflammatory cytokines such as IL-6, IL-1-beta, and TNF-alpha, as well as activation of pattern-recognition receptors causes secretion of G-CSF by macrophages, fibroblasts, endothelial cells, and other cell types participating in the local immune response. G-CSF binds to G-CSF receptors (G-CSFR) on mature neutrophils as well as immature neutrophil precursors. Downstream signaling via the JAK/STAT, MAPK, and PI3k/Akt pathways promotes survival, proliferation, and maturation. G-CSF signaling also promotes mobilization of neutrophils from the bone marrow into the bloodstream via downregulation of the SDF-1/CXCR4 pathway and has been shown to support neutrophil cytotoxic functions such as ROS production and phagocytosis (32). It is important to note that while endogenous G-CSF secretion results in mobilization and maturation of immature neutrophils within the bone marrow, exogenous G-CSF administration in high doses as used for stem cell mobilization in bone marrow transplant donors, can cause a transient increase of PMN-MDSCs in the blood (33).

The role of G-CSF in tumor development is an active area of investigation. Although G-CSF is used to treat neutropenia during chemotherapy administration, there are data to suggest that it can play a role in promoting tumor growth (**Table 1**). Analysis of patient samples has shown that many tumor cell types can produce G-CSF including colon, gastric, pancreas, hepatic, breast, ovarian, melanoma, and others (34). Although there is no definitive evidence that G-CSF predisposes to poor outcomes, high levels of G-CSF have been correlated with poor prognosis across solid malignancies. Several studies and case reports have shown poor outcomes in patients with GI malignancies and elevated G-CSF levels (34, 35). A retrospective analysis of patients with non-metastatic clear cell renal cell carcinoma treated with curative nephrectomy showed a significant association between high intra-tumoral G-CSF levels and reduced recurrence free survival. Notably, the association was stronger with late stage disease (36). A gene set variation analysis of breast cancer samples in the TCGA database identified a high-risk luminal breast cancer subtype with metastasis-promoting features that was enriched in neutrophils, highly expressed G-CSF, and was correlated with poor prognosis (37). Preclinical work in both *in vitro* and *in vivo* models of colon cancer has shown that treatment with G-CSF promotes tumor growth, while abrogation of the G-CSF/G-CSFR interaction leads to both decreased tumor growth as well as polarization of the TME towards an anti-tumorigenic state (38, 39).

**Table 1:** Summary of studies investigating G-CSF administration and outcomes.

Reference	Model system	Tissue source	Cancer type	Prior cancer-directed treatment	G-CSF source	Outcome
Fan et al (2018) (35) PMID: 29567938	Human	Patient tissue samples matched to adjacent mucosa, Human cell lines	Gastric	No prior treatment	Endogenous, Recombinant G-CSF	High intratumoral G-CSF level correlates with poor prognosis and G-CSFR expression correlated with lymph node metastasis *
Liu et at (2017) (36) PMID: 29050255	Human	Patient tissue samples	Clear Cell Renal Cell Carcinoma	No prior treatment	Endogenous	High G-CSF expression at time of nephrectomy associated with poor recurrence free survival
Guo et al (2019) (37) PMID: 30778902	Human	TCGA RNAseq data	Breast invasive/ lobular carcinoma	Unspecified	Endogenous, Recombinant GCSF	High G-CSF expression and neutrophil aggregation are associated with a high risk luminal breast cancer subtype with increased invasive capacity
Morris et al (2014) (38) PMID: 24448357	Human	Patient tissue samples, Human cell lines	Gastric and Colon Carcinoma	Unspecified	Endogenous, Recombinant GCSF	Intratumoral G-CSF induces proliferation and migration of tumor cells in vitro**
Morris et al (2015) (39) PMID: 26061815	Mouse	Carcinogen induced murine colon cancer tumors	Colon carcinoma	No prior treatment	Endogenous	G-CSF inhibition promotes anti tumorigenic TME and results in decreased tumor growth

G-CSF Outcomes:

*G-CSF protein level was upregulated from stage I to III then decreased in stage IV.

**G-CSF mRNA level was higher in stage 3 tumor samples that stage 2 or stage 4 tumors.

While this review will focus specifically on the effect of G-CSF on neutrophils in the TME, it is important to note that G-CSF exerts immunomodulatory effects on many innate and adaptive immune cells within the tumor. Some mechanisms of G-CSF-mediated modulation are generation of immunosuppressive ‘M2-like’ tumor-associated macrophages (TAMs) and myeloid-derived suppressor cells that inhibit cytotoxic T cell activity, and promote angiogenesis and proliferation (38). G-CSF has also been shown to disrupt the antigen-presenting function of dendritic cells within the TME and promote recruitment of regulatory T cells that inhibit cytotoxic T cell activity (40). Collectively, current literature suggests that G-CSF is part of an inflammatory cytokine milieu that alters the tumor immune landscape towards a more pro-tumorigenic state.

### G-CSF participates in an intratumoral signaling network that increases metastatic potential via pro-tumorigenic neutrophil polarization

**Figure 1:**
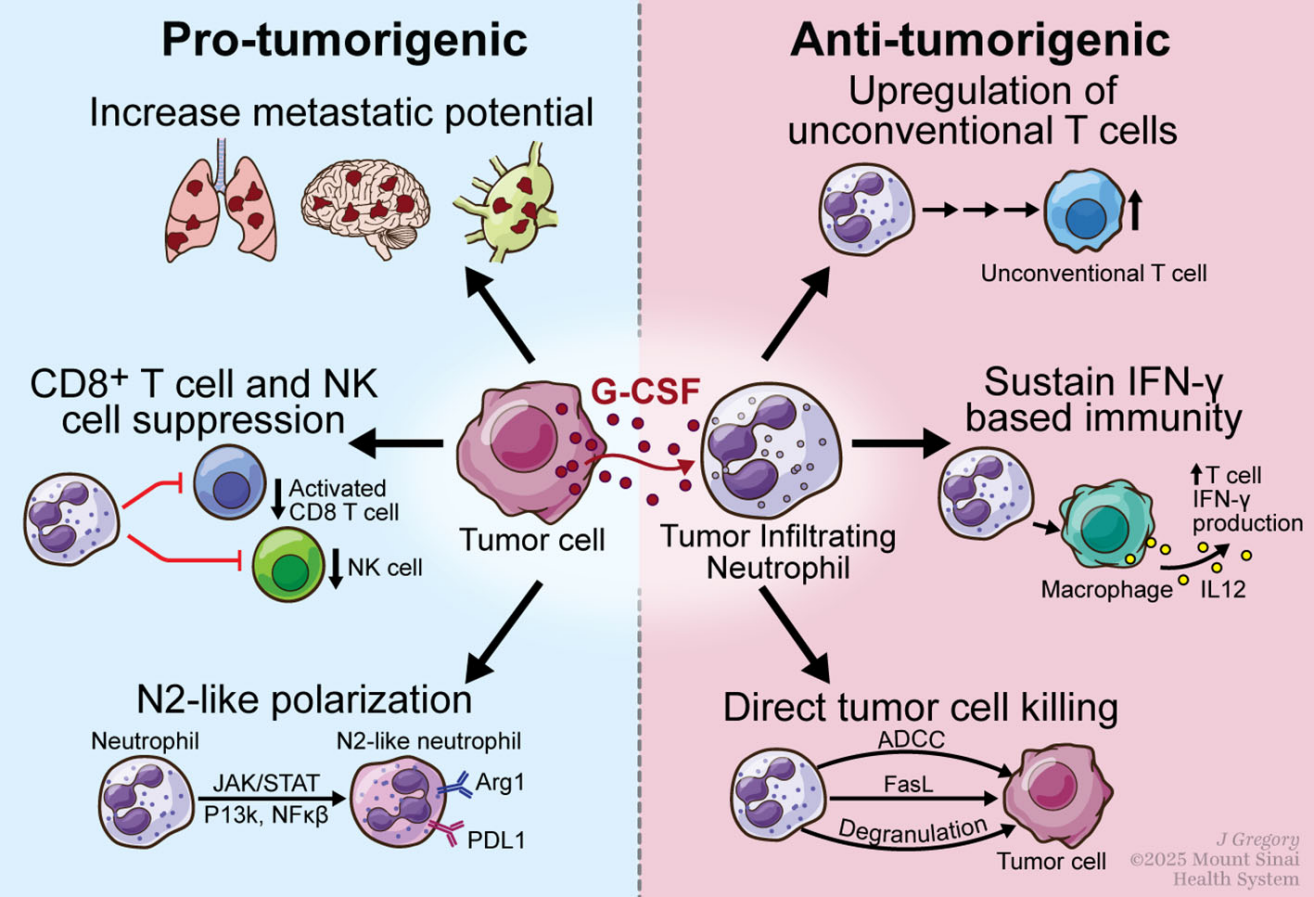
Pro-tumorigenic and anti-tumorigenic effects of G-CSF signaling on tumor infiltrating neutrophils. Pro-tumorigenic effects include promoting development of a premetastatic niche, polarization towards an N2-like phenotype, and suppression of CD8^+^ T cell and NK cell stimulation. Immunostimulatory or anti-tumorigenic effects include promoting T cell-based immunity via maintaining the IFN-γ pathway, upregulation of unconventional double negative T cells and direct tumor cell killing. The role of G-CSF is context dependent and is determined by the underlying immune state and composition of the TME.

One of the many effects of G-CSF within the TME is the polarization of neutrophils towards a pro-tumorigenic or ‘N2-like’ state (**Figure 1**, **Table 2**). Sheng et al. showed that G-CSF derived from human breast cancer cell lines prolonged the lifespan of healthy donor derived neutrophils via the PI3k/Akt and NF-kB pathways. These neutrophils also acquired new phenotypic features such as increased ability to stimulate cancer cell proliferation, migration, and invasion. G-CSF levels were also quantified in breast cancer surgical samples and found to correlate positively with TAN density, which separately correlated with poor prognosis and decreased progression-free survival (PFS) (41).

**Table 2:** Summary of reviewed studies investigating the role of G-CSF stimulated neutrophils within the TME including the observed net effect of neutrophils on tumor growth, classified as either pro-tumorigenic or anti-tumorigenic within the context of the study.

Effect	Reference	Model system	Tissue source	Cancer type	Prior cancer-directed treatment	G-CSF source	Neutrophil population
Pro-Tumorigenic	Sheng et al (2023) (41) PMID: 36801950	Human	Patient tissue samples, Human cell lines	Breast cancer, multiple subtypes	No prior therapy	Endogenous G-CSF, G-CSF overexpression, recombinant human G-CSF	Tumor Associated Neutrophils (TANS)
	Kowanetz et al (2010) (42) PMID: 21081700	Human Mouse	Mouse models, Human cell lines	Triple Negative breast cancer, Lung carcinoma, Melanoma	No prior therapy Docetaxel	Endogenous G-CSF, G-CSF overexpression, recombinant human and mouse G-CSF	Bone marrow derived Ly6G^+^Ly6C^+^ granulocytes
	Zhang et al (2020) (22) PMID: 32461334	Human Mouse	Mouse models, Human cell lines, Patient tissue samples	Breast Cancer, multiple subtypes	No prior therapy, Anti-CTLA4/ anti-PD1	Endogenous G-CSF, recombinant human and mouse GCSF	immunosuppressive Arg1^+^PDL1^+^ infiltrating neutrophils
	Coffelt et al (2015) (44) PMID: 25822788	Mouse	Mouse Models	Breast cancer	No prior therapy	Endogenous G-CSF, recombinant mouse G-CSF	Tumor induced neutrophils (TANs)
	Zou et al (2017) (45) PMID: 28432279	Mouse	Mouse Models	Hepatocellular carcinoma, Melanoma	No prior therapy	G-CSF overexpression	Tumor infiltrating neutrophils
Anti-Tumorigenic	Ponzetta et atl (2019) (46) PMID: 31257026	Mouse	Mouse Models	Sarcoma	No prior therapy	Endogenous G-CSF	Tumor infiltrating neutrophils
Context Dependent	Li et al (2020) (47) PMID: 32873795	Mouse	Mouse Models	Breast cancer	No prior therapy	Endogenous G-CSF, Recombinant mouse G-CSF	Tumor infiltrating neutrophils

Pro and Anti Tumorigenic Neutrophil Studies:

Consistent with the *in vitro* data, many studies suggest that G-CSF mediated neutrophil activation is associated with increased tumor metastatic potential (**Figure 1**, **Table 2**). Using a breast cancer mouse model, Kowanetz et al. showed that G-CSF induces tumor-infiltrating neutrophil migration to distant organs to establish a premetastatic niche. Treatment of breast cancer-carrying mice with G-CSF induced lung metastasis in previously non-metastatic mice. Furthermore, treatment with a G-CSF blocking antibody reduced lung metastasis in these mice (42). Another preclinical study, using both human cell lines and murine tumor models, found that EZH2-mediated G-CSF expression by breast cancer cells metastatic to the brain recruited immunosuppressive Arg1+/PDL1+ neutrophils into the brain, facilitating the growth of brain metastasis. Inhibiting G-CSF likewise resulted in less outgrowth of brain metastasis. The authors also showed that brain infiltrating neutrophils were positively correlated with brain metastasis in breast cancer patients (22). Similarly, a retrospective cohort study looked at the effect of G-CSF administration on brain metastasis in 121 stage IV breast cancer patients. Selection criteria included patients who did not have brain metastasis at the time of diagnosis and had received prior chemotherapy or targeted therapy. The study found that G-CSF users had a significantly greater incidence of brain metastasis compared to those who had not received G-CSF treatment, and that the risk increased in a dose-dependent manner. In a subgroup analysis, G-CSF use was associated with an increased risk of brain metastasis in patients who had not develop neutropenia after chemotherapy. However, in patients who did develop neutropenia after chemotherapy, G-CSF was associated with a decreased risk of brain metastasis. This finding highlights the importance of the immune state of the patient when treating with G-CSF and suggests that G-CSF may have disparate roles in different immune contexts (43).

The importance of the immune microenvironment on G-SCF signaling has become clearer as more work reveals the intertwined complexity of signaling cascades within the TME. Studies using murine tumor models have revealed that G-CSF secretion is part of an intra-tumoral signaling network that facilitates metastasis by repolarizing TANs towards an ‘N2-like’ protumorigenic phenotype. Coffelt et al. showed that TAN polarization occurs via an IL-1-beta/IL-17/G-CSF axis. IL-1-beta, likely produced by intra-tumoral macrophages, induced expression of IL-17 by γδ T cells. IL-17 in turn induced systemic upregulation of G-CSF, resulting in neutrophil expansion and polarization to a CD8+ T cell suppressive phenotype (**Figure 1**). Blockade of either IL-17 or G-CSF prevented neutrophil accumulation, suppression of T cell activation, and resulted in less pulmonary and lymph node metastasis. Interestingly, direct neutrophil depletion did not affect growth of the primary tumor but did result in less pulmonary and lymph node metastasis. Furthermore, neutrophil depletion decreased metastasis specifically in the early phase of the metastatic cascade, confirming their importance in early metastatic niche formation (44). Zou et al. then further expanded the network by showing that IL-35 stimulates intra-tumoral macrophages to secrete IL-1B, leading to the downstream effects described above. They also showed that intra-tumoral macrophages secrete IL-6, which together with G-CSF induced activation of the STAT3 and ERK pathways in neutrophils, leading to suppression of T cell activation via expression of iNOS (45). Taken together, these results suggest that chronic inflammation via sustained expression of proinflammatory cytokines within the TME results in elevated G-CSF expression, pro-tumorigenic neutrophil polarization, and increased metastatic potential (**Table 2**).

### Immunomodulatory effects of G-CSF are context dependent and G-CSF can promote anti-tumor immunity in the right context

Although the majority of the literature points towards a pro-tumorigenic role for G-CSF, there are also data demonstrating that G-CSF stimulated neutrophils have anti-tumorigenic functions (**Figure 1**, **Table 2**). Ponzetta et al. demonstrated that neutrophils were a critical component of the immune response to a carcinogen-induced sarcoma mouse model. Neutrophils were essential for production of IFNγ, a master regulator of the tumor immune response. Neutrophils sustained IFNγ-based immunity via macrophage secretion of IL-12, resulting in anti-tumor polarization of an unconventional double negative subset of T cells and decreased tumor growth. Conversely, when tumors were induced in C3f3R-mice, which lack the G-CSF receptor and are deficient in neutrophils, there was a marked reduction in the immune response characterized by lower IL-12 and IFNγ levels, pro-tumorigenic macrophage polarization, fewer and less activated unconventional T cells within the TME, and increased tumor growth (46).

Another study found that the effect of G-CSF on the TME is dependent on the underlying state of the immune system. Exogenous G-CSF augmented lung metastasis via neutrophil expansion in an immunocompetent breast cancer mouse model, however, it suppressed metastatic colonization in an immunocompromised mouse model lacking B, T, and NK cells. This was shown to occur via suppression of tumoricidal NK cells. The authors found that neutrophils have endogenous tumoricidal ability, however due to their suppression of the more cytotoxic NK cells, they exert a net pro-tumorigenic effect in an immunocompetent host. When NK cells are absent, the net effect of neutrophil infiltration is tumoricidal via their innate cytotoxic ability. As malignancy is a naturally immunocompromised state, the authors hypothesized that the effects of G-CSF may be similar in human cancer patients as in the immunocompromised mice (**Table 2**) (47).

### Functional impact of G-CSF signaling on immune checkpoint inhibitor therapy via neutrophil modulation

Given their immunosuppressive effects in the TME, N2-like neutrophils may negatively impact the efficacy of immunotherapy such as checkpoint blockade. The neutrophil-to-lymphocyte (NLR) ratio is a measurement of the absolute neutrophil count in comparison to the absolute lymphocyte count in peripheral blood. It is correlated with poor prognosis including worse progression-free survival and overall survival in patients treated with checkpoint inhibitors across cancer types (48). Consequently, it has been incorporated as an independent prognostic factor into the Gustave Roussy Immune Score (GRIm-score), a prognostic tool used to predict survival in patients enrolled on phase I clinical trials of immune checkpoint therapy (49). Although the NLR is an imperfect marker, as it does not take into account the different subtypes and activation states of granulocytes, it does suggest a detrimental effect of neutrophils in the context of malignancy.

As reviewed above, there are many potential mechanisms by which this effect may present, including suppression of CD8+ T cell activation, exclusion of T cells from the TME via NETs, regulatory T cell activation, creation of an immunosuppressive cytokine milieu, as well as other mechanisms not reviewed here. As G-CSF signaling promotes pro-tumorigenic neutrophil polarization, it may be hampering the efficacy of checkpoint inhibitor therapy via its modulation of neutrophil effector functions.

G-CSF exerts its effects on neutrophils through the JAK/STAT, MAPK, and PI3k/Akt pathways, which converge on activation of the Nf-KB and C/EBPB transcription factors, as reviewed in detail by Karagiannidis et al. (34). There have been many studies investigating the role of the PI3k/Akt pathway in PMN-MDSC function (50). The PI3k/Akt pathway is important for the immunosuppressive phenotype of PMN-MDSCs, and inhibiting this pathway results in abrogation of PMN-MDSC activity. One study showed that PI3k inhibition in a checkpoint blockade resistant mouse model of prostate cancer led to increased sensitivity to anti-PD1/anti-CTLA4 therapy (51). Another study showed enhanced response to PDL1 blockade when combined with PI3k inhibition in a mouse model of oral cancer. In this model, PI3k inhibition resulted in delay of tumor growth in T-cell inflamed tumors through decreased suppression of T-cell activity by granulocytic MDSCs. These MDSCs exhibited decreased expression of the immunosuppressive markers Arg1 and iNOS (52).

Overall, there is an abundance of pre-clinical literature suggesting that G-CSF may contribute to tumor growth, and especially to metastatic potential via pro-tumorigenic polarization of neutrophils. In mouse models, there is also data to suggest that G-CSF may impede checkpoint blockade through modulation of the neutrophil compartment within the TME. However, given a paucity of clinical data, there is as yet no clear indication that G-CSF administration is harmful to patients or that it worsens survival.

## G-CSF administration with chemotherapy and chemo-immunotherapy: clinical studies

### The good: exogenous G-CSF support with chemotherapy facilitates a safer and more consistent therapeutic course

At present, the role of G-CSF in patients receiving chemotherapy for solid malignancies is well established. Guidelines from the National Comprehensive Cancer Network (NCCN), American Society of Clinical Oncology (ASCO), and European Society for Medical Oncology (ESMO), all support primary G-CSF prophylaxis when the anticipated febrile neutropenia (FN) risk is ≥20% to reduce the risk of FN and enable timely and dose intense chemotherapy administration (53–55). However, the effect of G-CSF on chemo-immunotherapy regimens with regards to survival outcomes for patients is less well understood.

### The bad: net effect of G-CSF support during chemo-immunotherapy remains unclear

As treatment paradigms for solid malignancies continue to evolve to include the use of combined chemotherapy and immunotherapy, characterizing the impact of G-CSF on the TME in patients receiving chemo-immunotherapy is increasingly important. Recent clinical studies have begun to explore whether G-CSF may influence outcomes in patients receiving chemo-immunotherapy, and although these data are retrospective and heterogeneous, a consistent theme is the suggestion that G-CSF may interact with immune checkpoint blockade in clinically meaningful ways. Our review of the literature indicates that this interaction has thus far been studied almost exclusively in the treatment of lung cancer (**Table 3**).

**Table 3:** Summary of existing studies evaluating survival outcomes in patients being treated with chemo-immunotherapy and receiving G-CSF.

Reference study design	Cancer subtype	Chemo-immunotherapy regimen	G-CSF administered prophylactically vs therapeutically	Patient number	PFS (months) G-CSF vs NO G-CSF	OS (months) G-CSF vs NO G-CSF
Sun et al. (2025) (56) Retrospective analysis	Non-small cell lung cancer	4–6 cycles of standard platinum-based chemotherapy with PD-1 inhibitors + continue PD-1 inhibitor as maintenance therapy	**Primary prophylactic** PEG-rhG-CSF at a fixed dose of 6 mg, administered 48 hours after chemo-immunotherapy	220	12.6 vs 10.5 p = 0.99	30.3 vs not reached p = 0.85
Anpalakhan et al. (2023) (57) Retrospective analysis	Non-small cell lung cancer	First-line pembrolizumab plus platinum-based chemotherapy: • cisplatin–pemetrexed • carboplatin–pemetrexed • carboplatin–paclitaxel	**Primary prophylactic** non-pegylated G-CSF for at least 5 consecutive days (i.e., filgrastim 300–480 mcg or lenograstim 263 mcg subcutaneous daily), or pegylated GCSF for 1 day (i.e., pegfilgrastim 6 mg or lipegfilgrastim 6 mg), following each chemo-immunotherapy cycle starting from the first one	388	7.3 vs 8.4 p = 0.369	13.4 vs 12.6 p = 0.948
Ilhan et al. (2024) (58) Retrospective analysis	Extensive-stage small cell lung cancer	Carboplatin (5 AUC, day 1), etoposide (80–100 mg/m2, days 1, 2, and 3), and atezolizumab (1200 mg, day 1) every 21 days for 4–6 cycles, followed by atezolizumab maintenance therapy at a dose of 1200 mg every 21 days in the absence of disease progression	**Primary or secondary prophylaxis** with filgrastim, lenograstim, pegfilgrastim, and/or lipegfilgrastim were administered as G-CSF during chemo-immunotherapy, according to clinician preference	119	8.3 vs 6.8 p = 0.24	13.8 vs 10.6 p = 0.47
Tsukazi et al. (2024) (59) (60)	Extensive-stage small cell lung cancer	4 cycles of induction chemo-immunotherapy with carboplatin and etoposide plus either atezolizumab or durvalumab	**Therapeutic** administration of any G-CSF (≥ 1 dose of either filgrastim or pegfilgrastim)	65	**5.3 vs 9.5** **p = 0.005**	24.3 vs 16.4 p = 0.137

Primary G-CSF prophylaxis: use of G-CSF starting with the first cycle of chemo-immunotherapy to prevent neutropenia or febrile neutropenia. Secondary G-CSF prophylaxis: use of G-CSF in subsequent chemo-immunotherapy cycles after a patient has already experienced neutropenia or febrile neutropenia in a prior cycle without primary prophylaxis. Therapeutic G-CSF: use of G-CSF to treat established neutropenia or febrile neutropenia, rather than to prevent it.

Within the non-small cell lung cancer (NSCLC) population, Sun et al. (56) evaluated prophylactic pegylated G-CSF in patients with advanced NSCLC receiving first-line chemo-immunotherapy. While progression-free and overall survival (OS) were not significantly different between groups, outcomes signaled better OS rates in patients who did not receive treatment with G-CSF, raising the possibility that prophylaxis might not be neutral in the immunotherapy setting (56).

Earlier evidence from a large subgroup analysis of the SPINNAKER study by Anpalakhan et al. (57) similarly found no clear survival benefit associated with prophylactic G-CSF in advanced NSCLC. Notably, however, patients receiving G-CSF experienced a higher incidence of low-grade adverse events, including immune-related toxicities. This observation may suggest a potential immunomodulatory effect of G-CSF that may amplify immune activation without translating into improved antitumor efficacy (57).

Data in extensive-stage small cell lung cancer (ES-SCLC) were more inconsistent. A multicenter Turkish study by Ilhan et al. (58) reported comparable safety and survival outcomes with or without prophylactic G-CSF in patients treated with carboplatin/etoposide with atezolizumab, suggesting that G-CSF may be clinically permissive in this context (58).

In contrast, a contemporaneous Japanese multicenter study by Tsukazaki et al. (59) reported that therapeutic (rather than prophylactic) G-CSF administration was associated with significantly worse PFS and a reduced likelihood of durable responses to immunotherapy. Importantly, this negative association appeared specific to patients receiving immune checkpoint inhibitors, as G-CSF did not adversely affect outcomes in patients treated with chemotherapy alone (59). Updated long-term follow-up from the Japanese cohort reinforced these findings, consistently linking G-CSF exposure with inferior disease control despite adjustment for chemotherapy intensity and other confounders (60).

For now, the results reported by Tsukazaki et al. are the only data showing a statistically significant association between G-CSF use and decreased survival, specifically PFS. This may be because they used G-CSF to treat myelosuppression, whereas the other studies administered prophylactic G-CSF to prevent myelosuppression. In addition, these differences may indicate that patients treated in the Tsukazaki et al. cohort were sicker at the time of G-CSF administration and that this may have contributed to a worse PFS.

Thus far, none of the clinical data points to an improved PFS or OS in patients receiving G-CSF. Together, these clinical studies suggest that G-CSF may impair sustained immunotherapy benefit in a subset of patients, potentially through mechanisms discussed in the pre-clinical sections of this review. Taken as a whole, the emerging literature does not support a uniform survival benefit from G-CSF in chemo-immunotherapy regimens and instead raises concern that its use, particularly outside of strict guideline-based indications, may influence immune-mediated antitumor responses. These observations underscore the need for mechanistic studies and prospective trials to better define when, how, and in whom G-CSF can be safely integrated with cancer immunotherapy.

## Recommendations

The existing clinical data are not compelling in recommending against the use of G-CSF in patients being treated with chemo-immunotherapy. In addition to being discordant, the few available clinical studies have also all been conducted in patients with lung cancer subtypes, making it difficult to extrapolate their conclusions to other solid tumors. However, when coupled with pre-clinical data, they raise concern that G-CSF administration during chemo-immunotherapy might be associated with poorer survival outcomes, a reduced likelihood of durable responses to immunotherapy, and an increased risk of adverse effects.

Furthermore, this prompted us to investigate patterns of G-CSF administration in patients receiving chemo-immunotherapy, and we reviewed the data on the real-world use of G-CSF in the context of adherence to NCCN, ASCO, and ESMO guidelines. According to current literature, there is substantial discordance between expected guideline-recommended primary prophylaxis and clinically utilized (and reimbursed) practice. A number of studies found that G-CSF is underutilized in patients undergoing chemotherapy treatments associated with a high risk of FN, while being over utilized in patients with a low risk of FN, indicating only partial adherence to risk-stratified guidance both in the United States and Europe (61–63). It is worth noting that chemo-immunotherapy based regimens often have a lower risk of FN, and the pre-clinical data in this area should serve as a timely reminder to adhere strictly to the guidelines to ensure judicious use of G-CSF in these patients.

While we do not have compelling clinical data to suggest deviating from current guidelines when recommending use of G-CSF in patients receiving chemo-immunotherapy, the indication for G-CSF should be carefully considered, balancing the benefits of neutropenia prophylaxis against the potential for impaired antitumor immune responses and enhanced immunotherapy-related adverse effects.

### The unknown

The clinical utility of G-CSF in preventing FN and preserving chemotherapy dose intensity is unequivocal, and guidelines uniformly endorse its prophylactic use when the risk of neutropenia is high. However, long-term follow-up data have raised concerns regarding a modestly increased risk of therapy-related myeloid malignancies, although it remains uncertain whether this is a result of the increased leukemogenic potential of dose-dense chemotherapy versus a direct effect of G-CSF itself (or a combination of both).

Beyond its supportive role, G-CSF occupies a more ambiguous space within tumor immunobiology. Preclinical studies overwhelmingly highlight its capacity to skew neutrophils towards an ‘N2-like’ pro-tumorigenic state, promote metastatic niche formation, and blunt antitumor immune responses. Conversely, in select models, G-CSF-dependent neutrophils have demonstrated anti-tumor activity, suggesting that context—particularly the immune competence of the host and the composition of the tumor microenvironment—critically determines the functional outcome of G-CSF signaling.

Clinical data in the immunotherapy era remain sparse. Retrospective studies in lung cancer, where most of the work has been concentrated, suggest that G-CSF can be administered safely with chemo-immunotherapy in many patients. However, there are signals in the data that point to the possibility that G-CSF may attenuate immune checkpoint inhibitor efficacy, as reflected in shorter PFS and less durable responses. These observations are discordant, and differences in study design, patient selection, and timing of G-CSF administration (prophylaxis versus therapeutic administration) complicate interpretation.

The strong pre-clinical evidence and signals from the clinical data should prompt strict adherence to NCCN, ASCO, and ESMO guidelines for G-CSF administration. The balance between prophylaxis of life-threatening neutropenia and potential interference with anti-tumor immunity must be weighed on an individual basis, particularly in patients receiving first-line chemo-immunotherapy where maximizing response depth and durability is critical.

Moving forward, prospective trials with prespecified endpoints for survival, recurrence, and immune-related outcomes are needed to clarify the impact of G-CSF in patients receiving immunotherapy. Furthermore, integration of translational correlative studies—such as single-cell profiling, cytokine mapping, and functional assays of tumor-associated neutrophils—will be critical to link clinical observations with mechanistic insights. Until such data emerge, oncologists should continue to use G-CSF according to established guidelines for febrile neutropenia prophylaxis, while remaining aware of its potential pro-tumorigenic effects on the tumor microenvironment when used in conjunction with immune checkpoint blockade.

## References

[1] Mendoza-Valderrey A Dettmann E Hanes D Kessler D Danilova L Rau K . Immunogenomics and spatial proteomic mapping highlight distinct neuro-immune architectures in melanoma vs. non-melanoma-derived brain metastasis. BJC Rep. (2024) 2:38. doi: 10.1038/s44276-024-00060-y, PMID: 39516255 PMC11524107

[2] Shi Y Zhao X Zhou Y Zhang X . Neutrophil-to-lymphocyte ratio and platelet-to-lymphocyte ratio as predictive biomarkers for treatment response in primary advanced hypopharyngeal squamous cell carcinoma treated with chemoimmunotherapy. Clin Exp Med. (2025) 25:172. doi: 10.1007/s10238-025-01675-2, PMID: 40411692 PMC12103357

[3] Guida M Bartolomeo N Quaresmini D Quaglino P Madonna G Pigozzo J . Basal and one-month differed neutrophil, lymphocyte and platelet values and their ratios strongly predict the efficacy of checkpoint inhibitors immunotherapy in patients with advanced BRAF wild-type melanoma. J Transl Med. (2022) 20:159. doi: 10.1186/s12967-022-03359-x, PMID: 35382857 PMC8981693

[4] Zhou Y Shen G Zhou X Li J . Therapeutic potential of tumor-associated neutrophils: dual role and phenotypic plasticity. Signal Transduct Target Ther. (2025) 10:178. doi: 10.1038/s41392-025-02242-7, PMID: 40461514 PMC12134342

[5] Shi J Li J Wang H Li X Wang Q Zhao C . Single-cell profiling of tumor-associated neutrophils in advanced non-small cell lung cancer. Lung Cancer (Auckl). (2023) 14:85–99. doi: 10.2147/LCTT.S430967, PMID: 38025400 PMC10676108

[6] Xue R Zhang Q Cao Q Kong R Xiang X Liu H . Liver tumour immune microenvironment subtypes and neutrophil heterogeneity. Nature. (2022) 612:141–7. doi: 10.1038/s41586-022-05400-x, PMID: 36352227

[7] Awasthi D Sarode A . Neutrophils at the crossroads: unraveling the multifaceted role in the tumor microenvironment. Int J Mol Sci. (2024) 25:2929. doi: 10.3390/ijms25052929, PMID: 38474175 PMC10932322

[8] Fridlender J Kim S Kapoor V Cheng G Ling L . Polarization of tumor-associated neutrophil phenotype by TGF-beta: “N1” versus “N2” TAN. Cancer Cell. (2009) 16:183–94. doi: 10.1016/j.ccr.2009.06.017, PMID: 19732719 PMC2754404

[9] Qin F Liu X Chen J Huang S Wei W Zou Y . Anti-TGF-β attenuates tumor growth via polarization of tumor associated neutrophils towards an anti-tumor phenotype in colorectal cancer. J Cancer. (2020) 11:2580–92. doi: 10.7150/jca.38179, PMID: 32201528 PMC7066015

[10] Silvestre-Roig C Fridlender ZG Glogauer M Scapini P . Neutrophil diversity in health and disease. Trends Immunol. (2019) 40:565–83. doi: 10.1016/j.it.2019.04.012, PMID: 31160207 PMC7185435

[11] Antuamwine B Bosnjakovic R Hofmann-Vega F Wang X Theodosiou T . N1 versus N2 and PMN-MDSC: A critical appraisal of current concepts on tumor-associated neutrophils and new directions for human oncology. Immunol Rev. (2023) 314:250–79. doi: 10.1111/imr.13176, PMID: 36504274

[12] Cui C Chakraborty K Tang X Zhou G Schoenfelt K Becker K . Neutrophil elastase selectively kills cancer cells and attenuates tumorigenesis. Cell. (2021) 184:3163–3177.e21. doi: 10.1016/j.cell.2021.04.016, PMID: 33964209 PMC10712736

[13] Otten M Rudolph E Dechant M Tuk C Reijmers R Beelen R . Immature neutrophils mediate tumor cell killing via IgA but not IgG Fc receptors. J Immunol. (2005) 174:5472–80. doi: 10.4049/jimmunol.174.9.5472, PMID: 15843545

[14] Mysore V Cullere X Mears J Rosetti F Okubo K Liew P . FcγR engagement reprograms neutrophils into antigen cross-presenting cells that elicit acquired anti-tumor immunity. Nat Commun. (2021) 12:4791. doi: 10.1038/s41467-021-24591-x, PMID: 34373452 PMC8352912

[15] Bakema J Ganzevles S Fluitsma D Schilham M Beelen R Valerius T . Targeting FcαRI on polymorphonuclear cells induces tumor cell killing through autophagy. J Immunol. (2011) 187:726–32. doi: 10.4049/jimmunol.1002581, PMID: 21653835

[16] Lee J Han G Kyung M Lee S Kim TW Jung ST . Engineered neutrophil engagers overcome IgA limitations and reprogram resting neutrophils for cancer immunotherapy. J Biol Eng. (2025) 19:109. doi: 10.1186/s13036-025-00580-2, PMID: 41345694 PMC12679791

[17] Sun B Qin W Song M Liu L Yu Y Qi X . Neutrophil suppresses tumor cell proliferation via fas /fas ligand pathway mediated cell cycle arrested. Int J Biol Sci. (2018) 14:2103–13. doi: 10.7150/ijbs.29297, PMID: 30585273 PMC6299367

[18] Serrao KL Fortenberry JD Owens ML Harris FL Brown LA . Neutrophils induce apoptosis of lung epithelial cells via release of soluble Fas ligand. Am J Physiol Lung Cell Mol Physiol. (2001) 280:L298–305. doi: 10.1152/ajplung.2001.280.2.L298, PMID: 11159009

[19] Liu Y O'Leary C Wang L Bhatti T Dai N Kapoor V . CD11b+Ly6G+ cells inhibit tumor growth by suppressing IL-17 production at early stages of tumorigenesis. Oncoimmunology. (2016) 5:e1061175. doi: 10.1080/2162402X.2015.1061175, PMID: 26942073 PMC4760327

[20] Governa V Trella E Mele V Tornillo L Amicarella F Cremonesi E . The interplay between neutrophils and CD8+ T cells improves survival in human colorectal cancer. Clin Cancer Res. (2017) 23:3847–58. doi: 10.1158/1078-0432.CCR-16-2047, PMID: 28108544

[21] Zhang S Sun L Zuo J Feng D . Tumor associated neutrophils governs tumor progression through an IL-10/STAT3/PD-L1 feedback signaling loop in lung cancer. Transl Oncol. (2024) 40:101866. doi: 10.1016/j.tranon.2023.101866, PMID: 38128466 PMC10753083

[22] Zhang L Yao J Wei Y Zhou Z Li P Qu J . Blocking immunosuppressive neutrophils deters pY696-EZH2-driven brain metastases. Sci Transl Med. (2020) 12:eaaz5387. doi: 10.1126/scitranslmed.aaz5387, PMID: 32461334 PMC7948522

[23] Zhou S Zhou Z Hu Z Huang X Wang Z Chen E . Tumor-associated neutrophils recruit macrophages and T-regulatory cells to promote progression of hepatocellular carcinoma and resistance to sorafenib. Gastroenterology. (2016) 150:1646–1658.e17. doi: 10.1053/j.gastro.2016.02.040, PMID: 26924089

[24] Deryugina E Carré A Ardi V Muramatsu T Schmidt J Pham C . Neutrophil elastase facilitates tumor cell intravasation and early metastatic events. iScience. (2020) 23:101799. doi: 10.1016/j.isci.2020.101799, PMID: 33299970 PMC7702017

[25] Yang L Liu Q Zhang X Liu X Zhou B Chen J . DNA of neutrophil extracellular traps promotes cancer metastasis via CCDC25. Nature. (2020) 583:133–8. doi: 10.1038/s41586-020-2394-6, PMID: 32528174

[26] Albrengues J Shields M Ng D Park C Ambrico A Poindexter M . Neutrophil extracellular traps produced during inflammation awaken dormant cancer cells in mice. Science. (2018) 361:eaao4227. doi: 10.1126/science.aao4227, PMID: 30262472 PMC6777850

[27] El Rayes T Catena R Lee S Stawowczyk M Joshi N Fischbach C . Lung inflammation promotes metastasis through neutrophil protease-mediated degradation of Tsp-1. Proc Natl Acad Sci U S A. (2015) 112:16000–5. doi: 10.1073/pnas.1507294112, PMID: 26668367 PMC4703007

[28] Teijeira Á Garasa S Gato M Alfaro C Migueliz I Cirella A . CXCR1 and CXCR2 chemokine receptor agonists produced by tumors induce neutrophil extracellular traps that interfere with immune cytotoxicity. Immunity. (2020) 52:856–871.e8. doi: 10.1016/j.immuni.2020.03.001, PMID: 32289253

[29] Zhang Y Chandra V Riquelme Sanchez E Dutta P Quesada P . Interleukin-17-induced neutrophil extracellular traps mediate resistance to checkpoint blockade in pancreatic cancer. J Exp Med. (2020) 217:e20190354. doi: 10.1084/jem.20190354, PMID: 32860704 PMC7953739

[30] Eruslanov E Bhojnagarwala P Quatromoni J Stephen T Ranganathan A Deshpande C . Tumor-associated neutrophils stimulate T cell responses in early-stage human lung cancer. J Clin Invest. (2014) 124:5466–80. doi: 10.1172/JCI77053, PMID: 25384214 PMC4348966

[31] Mishalian I Bayuh R Levy L Zolotarov L Michaeli J Fridlender ZG . Tumor-associated neutrophils (TAN) develop pro-tumorigenic properties during tumor progression. Cancer Immunol Immunother. (2013) 62:1745–56. doi: 10.1007/s00262-013-1476-9, PMID: 24092389 PMC11028422

[32] Martin KR Wong HL Witko-Sarsat V Wicks IP . G-CSF - A double edge sword in neutrophil mediated immunity. Semin Immunol. (2021) 54:101516. doi: 10.1016/j.smim.2021.101516, PMID: 34728120

[33] Luyckx A Schouppe E Rutgeerts O Lenaerts C Fevery S Devos T . G-CSF stem cell mobilization in human donors induces polymorphonuclear and mononuclear myeloid-derived suppressor cells. Clin Immunol. (2012) 143:83–7. doi: 10.1016/j.clim.2012.01.011, PMID: 22341087

[34] Karagiannidis I Salataj E Said Abu Egal E Beswick EJ . G-CSF in tumors: Aggressiveness, tumor microenvironment and immune cell regulation. Cytokine. (2021) 142:155479. doi: 10.1016/j.cyto.2021.155479, PMID: 33677228 PMC8044051

[35] Fan Z Li Y Zhao Q Fan L Tan B Zuo J . Highly expressed granulocyte colony-stimulating factor (G-CSF) and granulocyte colony-stimulating factor receptor (G-CSFR) in human gastric cancer leads to poor survival. Med Sci Monit. (2018) 24:1701–11. doi: 10.12659/msm.909128, PMID: 29567938 PMC5880331

[36] Liu Z Zhu Y Wang Y Fu Q Fu H Wang Z . Prognostic value of granulocyte colony-stimulating factor in patients with non-metastatic clear cell renal cell carcinoma. Oncotarget. (2017) 8:69961–71. doi: 10.18632/oncotarget.19540, PMID: 29050255 PMC5642530

[37] Guo L Chen G Zhang W Zhou L Xiao T Di X . A high-risk luminal A dominant breast cancer subtype with increased mobility. Breast Cancer Res Treat. (2019) 175:459–72. doi: 10.1007/s10549-019-05135-w, PMID: 30778902 PMC6533414

[38] Morris K Castillo E Ray A Weston L Nofchissey R Hanson J . Anti-G-CSF treatment induces protective tumor immunity in mouse colon cancer by promoting protective NK cell, macrophage and T cell responses. Oncotarget. (2015) 6:22338–47. doi: 10.18632/oncotarget.4169, PMID: 26061815 PMC4673167

[39] Morris K Khan H Ahmad A Weston L Nofchissey R Pinchuk I . G-CSF and G-CSFR are highly expressed in human gastric and colon cancers and promote carcinoma cell proliferation and migration. Br J Cancer. (2014) 110:1211–20. doi: 10.1038/bjc.2013.822, PMID: 24448357 PMC3950854

[40] Mouchemore KA Anderson RL . Immunomodulatory effects of G-CSF in cancer: Therapeutic implications. Semin Immunol. (2021) 54:101512. doi: 10.1016/j.smim.2021.101512, PMID: 34763974

[41] Sheng Y Peng W Huang Y Cheng L Meng Y Kwantwi L . Tumor-activated neutrophils promote metastasis in breast cancer via the G-CSF-RLN2-MMP-9 axis. J Leukoc Biol. (2023) 113:383–99. doi: 10.1093/jleuko/qiad004, PMID: 36801950

[42] Kowanetz M Wu X Lee J Tan M Hagenbeek T Qu X . Granulocyte-colony stimulating factor promotes lung metastasis through mobilization of Ly6G+Ly6C+ granulocytes. Proc Natl Acad Sci U S A. (2010) 107:21248–55. doi: 10.1073/pnas.1015855107, PMID: 21081700 PMC3003076

[43] Tai YS Leung JH Wang SY Leung HWC Chan ALF . Association of granulocyte colony-stimulating factor treatment with risk of brain metastasis in advanced stage breast cancer. Int J Mol Sci. (2024) 25:10756. doi: 10.3390/ijms251910756, PMID: 39409083 PMC11477282

[44] Coffelt S Kersten K Doornebal C Weiden J Vrijland K Hau C . IL-17-producing γδ T cells and neutrophils conspire to promote breast cancer metastasis. Nature. (2015) 522:345–8. doi: 10.1038/nature14282, PMID: 25822788 PMC4475637

[45] Zou J Qin J Li Y Wang Y Li D Shu Y . IL-35 induces N2 phenotype of neutrophils to promote tumor growth. Oncotarget. (2017) 8:33501–14. doi: 10.18632/oncotarget.16819, PMID: 28432279 PMC5464885

[46] Ponzetta A Carriero R Carnevale S Barbagallo M Molgora M Perucchini C . Neutrophils driving unconventional T cells mediate resistance against murine sarcomas and selected human tumors. Cell. (2019) 178:346–360.e24. doi: 10.1016/j.cell.2019.05.047, PMID: 31257026 PMC6630709

[47] Li P Lu M Shi J Hua L Gong Z Li Q . Dual roles of neutrophils in metastatic colonization are governed by the host NK cell status. Nat Commun. (2020) 11:4387. doi: 10.1038/s41467-020-18125-0, PMID: 32873795 PMC7463263

[48] Faget J Peters S Quantin X Meylan E Bonnefoy N . Neutrophils in the era of immune checkpoint blockade. J Immunother Cancer. (2021) 9:e002242. doi: 10.1136/jitc-2020-002242, PMID: 34301813 PMC8728357

[49] Bigot F Castanon E Baldini C Hollebecque A Carmona A Postel-Vinay S . Prospective validation of a prognostic score for patients in immunotherapy phase I trials: The Gustave Roussy Immune Score (GRIm-Score). Eur J Cancer. (2017) 84:212–8. doi: 10.1016/j.ejca.2017.07.027, PMID: 28826074

[50] Ibrahim A Mohamady Farouk Abdalsalam N Liang Z Kashaf Tariq H Li R Afolabi O L . MDSC checkpoint blockade therapy: a new breakthrough point overcoming immunosuppression in cancer immunotherapy. Cancer Gene Ther. (2025) 32:371–92. doi: 10.1038/s41417-025-00886-9, PMID: 40140724 PMC11976280

[51] Lu X Horner J Paul E Shang X Troncoso P Deng P . Effective combinatorial immunotherapy for castration-resistant prostate cancer. Nature. (2017) 543:728–32. doi: 10.1038/nature21676, PMID: 28321130 PMC5374023

[52] Davis R Moore E Clavijo P Friedman J Cash H Chen Z . Anti-PD-L1 efficacy can be enhanced by inhibition of myeloid-derived suppressor cells with a selective inhibitor of PI3Kδ/γ. Cancer Res. (2017) 77:2607–19. doi: 10.1158/0008-5472.CAN-16-2534, PMID: 28364000 PMC5466078

[53] Ahmed I Bachiashvili K Baird J . NCCN Guidelines Version 3.2026 Hematopoietic Growth Factors (2025). Available online at: https://www.nccn.org/home/member- (Accessed December 13, 2025).

[54] Smith T Bohlke K Lyman G Carson K Crawford J Cross S . Recommendations for the use of WBC growth factors: American society of clinical oncology clinical practice guideline update. J Clin Oncol. (2015) 33:3199–212. doi: 10.1200/JCO.2015.62.3488, PMID: 26169616

[55] Crawford J Caserta C Roila F . ESMO Guidelines Working Group. Hematopoietic growth factors: ESMO Clinical Practice Guidelines for the applications. Ann Oncol. (2010) 21:v248–51. doi: 10.1093/annonc/mdq195, PMID: 20555091

[56] Sun L Tian Y Zhang S Huang L Ma J Han C . Impact of prophylactic use of PEG-rhG-CSF on first-line immunochemotherapy in advanced NSCLC: A cohort study. JTO Clin Res Rep. (2025) 6:100780. doi: 10.1016/j.jtocrr.2024.100780, PMID: 39877027 PMC11773054

[57] Anpalakhan S Huddar P Behrouzi R Signori A Cave J Comins C . The effects of GCSF primary prophylaxis on survival outcomes and toxicity in patients with advanced non-small cell lung cancer on first-line chemoimmunotherapy: A sub-analysis of the spinnaker study. Int J Mol Sci. (2023) 24:1746. doi: 10.3390/ijms24021746, PMID: 36675262 PMC9867035

[58] Ilhan Y Ucar G Baser M Guzel H Efil S Demir B . Efficacy and safety of G-CSF prophylaxis in patients with extensive-stage small cell lung cancer receiving chemoimmunotherapy. Expert Opin Pharmacother. (2024) 25:1555–63. doi: 10.1080/14656566.2024.2391007, PMID: 39115275

[59] Tsukazaki Y Ogino H Okano Y Kakiuchi S Harada S Toyoda Y . Granulocyte colony-stimulating factor has the potential to attenuate the therapeutic efficacy of chemo-immunotherapy for extensive-stage small-cell lung cancer. Int J Clin Oncol. (2024) 29:1451–60. doi: 10.1007/s10147-024-02586-0, PMID: 39009900

[60] Tsukazaki Y Ogino H Okano Y Kakiuchi S Harada S Toyoda Y . The conflicting impacts of G-CSF on the therapeutic efficacy of chemoimmunotherapy for extensive stage small cell lung cancer. In: 2024 world conference on lung cancer. J Thorac Oncol. (2024) 19:S676. doi: 10.1016/j.jtho.2024.09.1278

[61] Barnes G Pathak A Schwartzberg L . G-CSF utilization rate and prescribing patterns in United States: associations between physician and patient factors and GCSF use. Cancer Med. (2014) 3:1477–84. doi: 10.1002/cam4.344, PMID: 25410813 PMC4298373

[62] Van Ryckeghem F Haverbeke C Wynendaele W Jerusalem G Somers L Van den Broeck A . Real-world use of granulocyte colony-stimulating factor in ambulatory breast cancer patients: a cross-sectional study. Support Care Cancer. (2019) 27:1099–108. doi: 10.1007/s00520-018-4399-3, PMID: 30099601

[63] Baig H Somlo B Eisen M Stryker S Bensink M Morrow PK . Appropriateness of granulocyte colony-stimulating factor use in patients receiving chemotherapy by febrile neutropenia risk level. J Oncol Pharm Pract. (2019) 25:1576–85. doi: 10.1177/1078155218799859, PMID: 30200842 PMC6716357

